# WireGC-Former: Surface defect segmentation method of steel wire ropes based on 3D point clouds

**DOI:** 10.1371/journal.pone.0356272

**Published:** 2026-08-14

**Authors:** Chengjun Wang, Junyi Li, Qing Liu, Chang Zhao, Yuliang Li, Yanqiu Zhao

**Affiliations:** 1 School of Artificial Intelligence, Anhui University of Science and Technology, Huainan, Anhui, China; 2 School of Artificial Intelligence, Anhui Polytechnic University, Wuhu, Anhui, China; 3 Anhui Wanbei Coal-Electricity Group Co., Ltd., Suzhou, Anhui, China; Hohai University, CHINA

## Abstract

Three-dimensional point clouds can accurately represent the spatial geometric information of industrial product surfaces, offering significant value in small-target defect segmentation tasks. To address the scarcity of high-quality datasets in existing research on industrial defect point cloud segmentation, as well as the challenges posed by the small scale, sparse distribution, and complex morphology of wire rope surface defects, this paper constructs a wire rope defect point cloud dataset, WireRope3D, covering three typical types of defects: wear, wire protrusion, and broken wire. Furthermore, we propose a point cloud segmentation method named WireGC-Former (Wire Rope Graph Convolution Transformer), which integrates a graph convolutional network with a Transformer. The method enhances the model’s ability to perceive local geometric structures and defect boundaries through an edge feature extraction module; improves the representation of local spatial relationships by incorporating a spatial attention module that fuses absolute coordinate information with relative positional encoding; integrates multi‑level local and global features via a feature fusion module; and models long‑range dependencies with a feature attention module, thereby achieving precise segmentation of small‑target defects on wire rope surfaces. Experimental results show that WireGC-Former achieves a mean Intersection over Union (mIoU) of 80.32% on the WireRope3D dataset, demonstrating the effectiveness of the proposed method for small‑target defect segmentation on wire rope surfaces. The presented method can provide a reliable data foundation and technical support for refined detection and subsequent quantitative analysis of wire rope surface defects, and serve as a reference for point cloud semantic segmentation research in complex industrial scenarios.

## 1. Introduction

As a core force-transmitting component in hoisting machinery, mine hoisting systems, and various load‑bearing equipment, the service condition of wire ropes directly determines the reliability and safety of equipment operation [1,2]. With the extension of service life, various types of damage, such as wear, wire protrusion, and broken wire, inevitably occur on the rope surface. These defects lead to degradation of load‑bearing capacity and shorten the service life; in extreme cases, they can induce equipment failure and even safety accidents. Therefore, developing high‑precision defect detection and segmentation methods for wire rope surfaces has become a critical issue that urgently needs to be addressed in engineering practice.

Currently, wire rope defect detection methods mainly include manual visual inspection, electromagnetic nondestructive testing [3–6], and image‑based visual inspection [7]. However, each type of method has different degrees of limitations in terms of efficiency, accuracy, and environmental adaptability. Manual inspection is labor‑intensive and inefficient, making it difficult to meet the demands of large‑scale continuous inspection. The basic principle of electromagnetic testing is to apply magnetic excitation to the wire rope and identify internal and surface defects (e.g., broken wire, wear) by capturing changes in the magnetic flux leakage field. For example, Tan et al. [8] proposed an under‑saturated magnetic excitation scheme to achieve effective identification of rope damage; Mazurek et al. [9] introduced tunnel magnetoresistance (TMR) sensors to improve the detection sensitivity of damage signals. However, in complex working conditions such as mines with strong electromagnetic interference, the magnetic flux leakage signal is susceptible to noise‑induced attenuation [10]. Moreover, the information obtained by electromagnetic testing is essentially one‑dimensional, offering limited capability to characterize the surface geometry of defects, which hinders fine‑grained spatial description and quantitative analysis. Visual inspection methods use image processing and deep learning algorithms to automatically identify surface damage on wire ropes. Huang et al. [11] employed convolutional neural networks (CNNs) to automatically learn feature representations from surface images, enabling defect classification. Zhou et al. [12] further proposed the WR‑IPDCNN framework, applying a deep convolutional network to end‑to‑end identification of wire rope surface damage. Nevertheless, visual inspection methods heavily depend on the acquisition environment—non‑uniform illumination or cluttered backgrounds can degrade image quality, thereby limiting detection accuracy and robustness.

In recent years, with the continuous advancement of 3D laser scanning technology, the cost of point cloud data acquisition has significantly decreased, while spatial resolution has steadily improved, making large‑scale acquisition of high‑precision point cloud data a reality. Especially in industrial scenes with complex illumination, highly reflective metal surfaces, and the need for fine‑grained morphology description, laser point clouds exhibit stronger environmental robustness and higher spatial expression accuracy than traditional vision‑based methods, providing strong support for precise localization and automatic identification of complex surface defects [13,14]. In this context, Wang et al. [15] first introduced laser point cloud data into the field of wire rope inspection, proposing the PMAFNet to achieve region‑level detection of broken wire and wear defects on wire rope surfaces, thereby verifying the feasibility and effectiveness of using point cloud data for surface damage identification. However, region‑level detection only provides coarse localization and category judgment, failing to accurately describe defect boundaries at the point level. To address this, Liu et al. [16] advanced the task to point‑wise semantic segmentation, proposing the Point S‑GS Former network that integrates spatial‑domain and graph‑spectral‑domain features. By using graph Fourier transform to capture global structural patterns, they achieved more refined point‑level representation of defect geometric contours. These works have promoted the evolution of laser‑point‑cloud‑based wire rope defect analysis from coarse‑grained localization to fine‑grained segmentation. Nevertheless, existing methods still struggle to achieve an ideal balance between fully exploiting local geometric priors and efficiently modeling global context.

The above studies indicate that laser‑point‑cloud‑based wire rope defect segmentation has gradually moved from coarse‑grained localization to fine‑grained representation. However, existing methods still suffer from an imbalance between the utilization of local geometric priors and the modeling of global context information. Specifically, pure spatial‑domain methods have limited ability to represent the anisotropy of local neighborhood geometry, while introducing spectral‑domain analysis, although it compensates for the lack of global perception, comes with high computational overhead due to the eigen‑decomposition of the graph Laplacian matrix, restricting the practicality of models on large‑scale point clouds. Moreover, the non‑uniform characteristics of wire rope surface defects in terms of scale and density distribution impose higher requirements on effective cross‑level feature fusion.

To address the above limitations, this paper proposes WireGC-Former, a point cloud segmentation network that combines graph convolution with Transformer-based contextual modeling for point-wise segmentation of wire-rope surface defects. Unlike DGCNN, which primarily propagates local edge-difference features, and Point Transformer, which mainly employs position-aware attention to model point relationships, WireGC-Former establishes a task-specific local-to-global feature propagation process for defects characterized by small size, sparse distribution, complex morphology, and ambiguous boundaries.

The technical contribution of WireGC-Former does not lie in introducing a new EdgeConv or Transformer operator. Instead, it lies in coordinating local geometric enhancement, spatial position guidance, cross-layer feature fusion, and global contextual calibration within a unified architecture. Specifically, the Edge Feature Extraction Module (EFEM) captures local geometric discontinuities and boundary variations through neighborhood edge features. The Spatial Attention Module (SAM) jointly encodes relative positions and absolute coordinates and uses learnable attention weights to adaptively aggregate neighborhood information. The Feature Fusion Module (FFM) integrates and compresses multi-level local features into a compact representation before global modeling, thereby reducing feature redundancy and computational overhead. Finally, the Feature Attention Module (FAM) models long-range dependencies and calibrates the fused local representation using Transformer-based self-attention. Through this sequential feature propagation process, the network preserves weak local defect cues while exploiting global contextual information to distinguish sparse defect points from the dominant normal background.

The main contributions of this paper are summarized as follows:

(1) We construct a wire rope surface defect point cloud dataset, WireRope3D, covering three typical defects (wear, wire protrusion, and broken wire) and containing 2,916 high‑precision samples, providing a standardized data foundation for wire rope defect point cloud segmentation research.
(2) We propose a point cloud segmentation network, WireGC‑Former, that combines a GCN with a Transformer for point‑level semantic segmentation of wire rope surface defects. The network consists of an edge feature extraction module, a spatial attention module, a feature fusion module, and a feature attention module, forming a local‑global collaborative modeling framework that enhances the joint representation capability of local geometric details and global context relationships.
(3) Experimental results on the WireRope3D dataset demonstrate that the proposed method outperforms several representative baseline models in overall segmentation performance, and ablation studies verify the effectiveness of each key module.

The remainder of this paper is organized as follows. Section 2 introduces related research on point cloud defect segmentation, including methods based on local geometric modeling and those based on attention mechanisms. Section 3 details the overall architecture and each component module of WireGC‑Former. Section 4 describes the dataset construction process, experimental setup, and analysis of experimental results. Section 5 concludes the paper and discusses future research directions.

## 2. Related works

### 2.1. Point cloud-based segmentation methods

Point cloud-based segmentation methods can be primarily categorized into three types according to their feature modeling approaches: point-based methods, graph-based methods, and attention mechanism-based methods.

Point-based methods perform feature learning directly on raw point clouds. Qi et al. [17] proposed PointNet, the first end-to-end deep learning framework for point clouds, which extracts per-point features through shared multilayer perceptrons and achieves permutation invariance of unordered point sets via symmetric functions. Building upon this, Qi et al. [18] further introduced PointNet++, which incorporates hierarchical sampling and grouping strategies to enhance the capture of local spatial structures, demonstrating superior performance in dense point cloud and fine-grained semantic segmentation tasks. Additionally, Thomas et al. [19] proposed KPConv, which performs convolution operations on local neighborhoods using kernel point convolution operators, effectively improving the representation capability of geometric features. The advantage of such methods lies in their ability to directly utilize the original three-dimensional coordinate information of point clouds; however, they still exhibit limitations in modeling long-range dependencies.

Graph-based methods transform point clouds into graph structures and characterize local geometric topology by explicitly modeling adjacency relationships between points. Wang et al. [20] proposed DGCNN, which employs dynamic graph convolution to update adjacency relationships in real-time within the feature space, enabling adaptive modeling of geometric structures and significantly enhancing the capture of local detail features such as edges and surface deformations. Bahreini et al. [21] further integrated multimodal information, including normal vectors and color, into graph modeling, thereby improving the representation capability of geometric features at defect edges. Nevertheless, graph-based methods encounter computational overhead challenges associated with the construction and updating of adjacency matrices when processing large-scale point clouds, and their scalability remains to be improved.

The aforementioned methods based on local geometric modeling exhibit advantages in defect edge perception and local structural representation. However, for sparsely distributed small-target defects, relying solely on local neighborhood features is insufficient to adequately model their semantic associations within the global point cloud. With the widespread application of Transformer architectures in vision tasks, attention mechanism-based methods have been introduced into the field of point cloud segmentation. Representative studies, such as Point Transformer [22], introduce positional encoding and attention weights to adaptively capture long-range dependencies arising from irregular sampling in point clouds, thereby achieving global context modeling capability. Lu et al. [23] proposed a novel hierarchical framework that combines convolution operations with Transformer architectures; however, the generalization capability of the model requires further validation. Nonetheless, existing attention-based methods remain susceptible to interference from background noise and irrelevant neighborhood features in industrial defect scenarios, exhibiting limited capability in stably identifying small-scale, sparsely distributed defects. Therefore, how to effectively integrate local geometric feature extraction and global context modeling, so that the network has the sensitivity to defect edges and the ability to express long-distance dependence, is a key issue that needs to be solved urgently in the current research on industrial point cloud segmentation.

### 2.2. Point cloud defect detection in industrial scenarios

Point cloud-based defect detection employs deep neural networks to automatically learn three-dimensional geometric features, thereby enabling efficient identification and localization of industrial surface defects. According to differences in learning paradigms, existing methods can be primarily categorized into three types: supervised learning methods, self-supervised learning methods, and unsupervised learning methods.

In the realm of self-supervised learning, Bergmann and Sattlegger [24] proposed a teacher-student network framework based on deep geometric descriptors. A teacher network is pre-trained in a self-supervised manner to extract local geometric features, and a student network is trained to fit the teacher's output on normal samples. During testing, the regression error between the outputs of the two networks is used to assign an anomaly score to each point, enabling precise defect localization. Li et al. [25] proposed a self-supervised method based on iterative mask reconstruction. During the training phase, the network learns the distribution characteristics of normal point clouds through repeated masking and reconstruction operations. In the testing phase, defect regions are localized by comparing the point cloud after multiple iterative reconstructions with the original point cloud and utilizing the spatial distribution of reconstruction errors. Ye et al. [26] guided the model to learn features of anomalous regions by predicting point offsets in pseudo-anomalous point clouds, and precisely delineated defect boundaries through the gradient variations of these offsets.

In the field of unsupervised learning, Zhao et al. [27] proposed a unified memory bank framework that fuses local coordinate features with global PointMAE features. The distribution of features with different magnitudes is balanced through ranking normalization, and anomaly scores are calculated by measuring the distance between features and the memory bank. Liu et al. [28] introduced the Real3D-AD method, which combines coordinate features and PointMAE features to construct a dual memory bank, capturing the local localization attributes and global structural information of point clouds respectively, thereby achieving defect detection and localization. Liu et al. [29] further proposed the Uni-3DAD method, which integrates a PatchCore detection module based on FPFH features with a reconstruction-based missing region detection module. The anomaly scores from both branches are fused via OCSVM to enhance the detection capability for small defect regions from the two dimensions of geometric features and structural integrity. Additionally, Cao et al. [30] proposed a multimodal fusion strategy that combines handcrafted FPFH 3D descriptors with 2D semantic features generated from multi-view projections. Through feature alignment aggregation and memory bank matching, this approach enhances sensitivity to minute defects. Zhou et al. [31] proposed the R3D-AD method, which utilizes a diffusion model to reconstruct anomalous point clouds. Training data is augmented by simulating defects via a Patch-Gen strategy, and shape embeddings extracted by PointNet are injected during the reverse diffusion process. Defect localization is achieved by measuring the spatial distance between the reconstructed point cloud and the original point cloud. Zhu et al. [32] proposed a high-resolution point cloud anomaly detection framework based on group-level feature contrastive learning. By optimizing feature cluster distribution and dynamically focusing on potentially anomalous regions using geometric information, this framework effectively addresses the challenge of capturing minute defects in high-resolution point clouds.

In summary, point cloud-based defect detection methods fully leverage three-dimensional geometric information, are unaffected by lighting conditions, and are suitable for defect analysis on complex curved surfaces. However, existing methods are predominantly oriented toward general industrial scenarios. For specific targets such as steel wire ropes, which possess complex helical structures and multi-scale defect characteristics, there remains considerable room for improvement in both detection accuracy and computational efficiency.

## 3. Methodology

**Fig 1:**
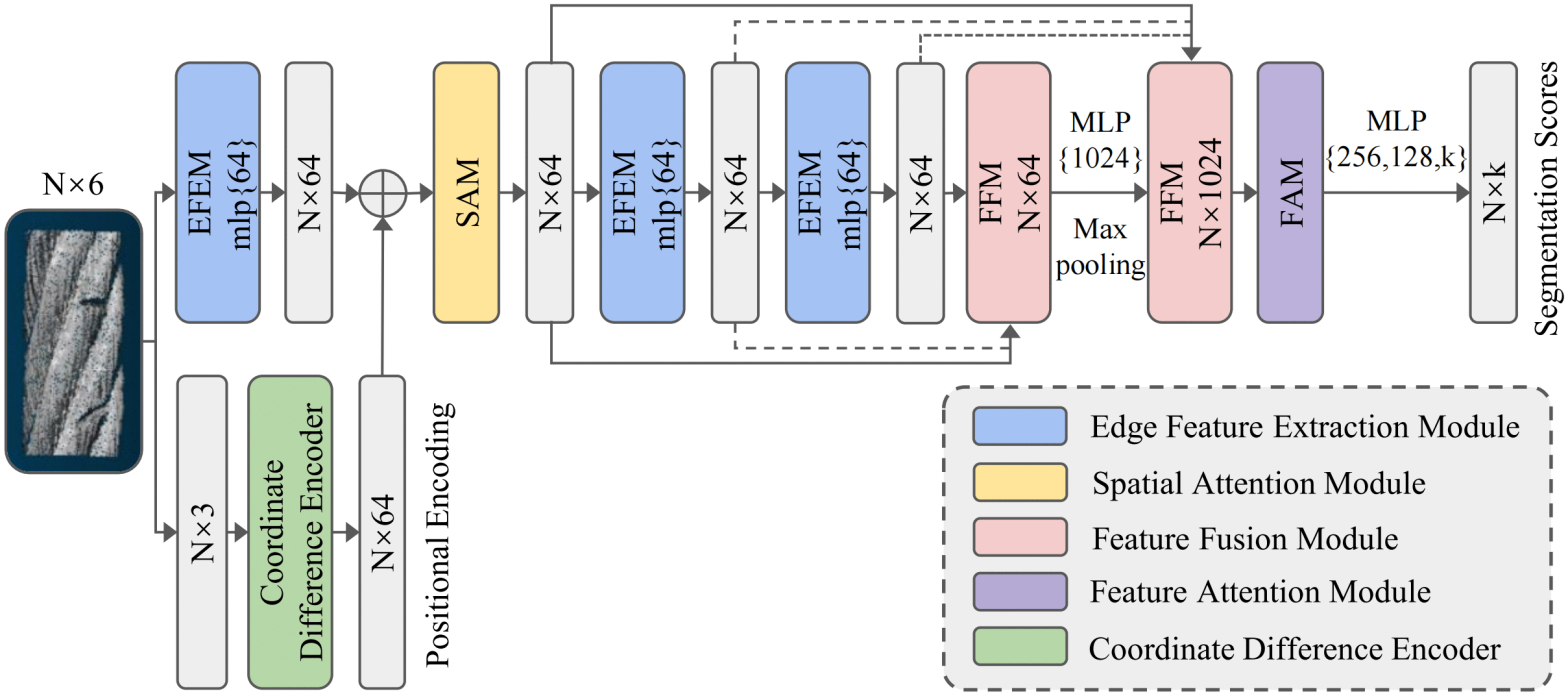
Overall architecture of WireGC-Former.

To address the limitations of existing methods based on local geometric modeling in global dependency modeling, as well as the insufficient local geometric representation of attention-based methods in complex industrial defect scenarios, this paper proposes a point cloud segmentation network architecture named WireGC-Former (Wire Rope Graph Convolution Transformer), which combines GCN and Transformer, as shown in Fig 1. The input to the network is raw point cloud data of size N × 6, where N is the number of points in the input point cloud, and the six channels correspond to 3D coordinates (XYZ) and color information (RGB). The overall network consists of four core modules: the Edge Feature Extraction Module (EFEM), the Spatial Attention Module (SAM), the Feature Fusion Module (FFM), and the Transformer-based Feature Attention Module (FAM).

First, EFEM is used to extract neighborhood feature information from the point cloud. Simultaneously, coordinate differences are computed from the first three coordinate channels for positional encoding, and the encoding results are concatenated with the neighborhood feature vectors. The features are then fed into SAM, where learnable attention scores are normalized using Softmax and employed to perform weighted neighborhood aggregation. A residual connection is subsequently introduced to preserve the original point-wise features. After the features from different layers are integrated through FFM, they are passed to the FAM module to model long-range dependencies. Finally, the model performs semantic segmentation of the point cloud by concatenating the global descriptor with the local descriptor of each point (the latter fuses distance-based positional encoding with neighborhood features), and outputs a four-class semantic scores for background, wear, wire protrusion, and broken wire.

The core idea of this network design is as follows: EFEM and SAM are used to fully extract local geometric features, FFM achieves efficient fusion of multi-scale features, and finally FAM models global context dependencies. This hierarchical feature modeling strategy, progressing from local to global, enables the network to maintain sensitivity to small-target defects while effectively leveraging global context information for semantic discrimination.

### 3.1. Edge feature extraction module

In terms of local feature extraction from point clouds, the EdgeConv module proposed by DGCNN effectively models local geometric relationships by dynamically constructing a K-nearest neighbor (KNN) graph and computing edge features between the central point and its neighboring points. Compared with methods that only perform independent mapping on single point features, EdgeConv simultaneously considers both the point itself and the relative variations within its neighborhood, thus offering significant advantages in boundary detail characterization, local structure representation, and anomaly region identification. For wire rope surface defect point clouds, defects such as wear, wire protrusions, and broken wires often manifest as disruptions in local geometric continuity or abrupt changes in spatial morphology. Therefore, explicitly describing the differences between a point and its neighborhood through edge features helps enhance the model's sensitivity to defect boundaries and anomalous regions.

**Fig 2:**
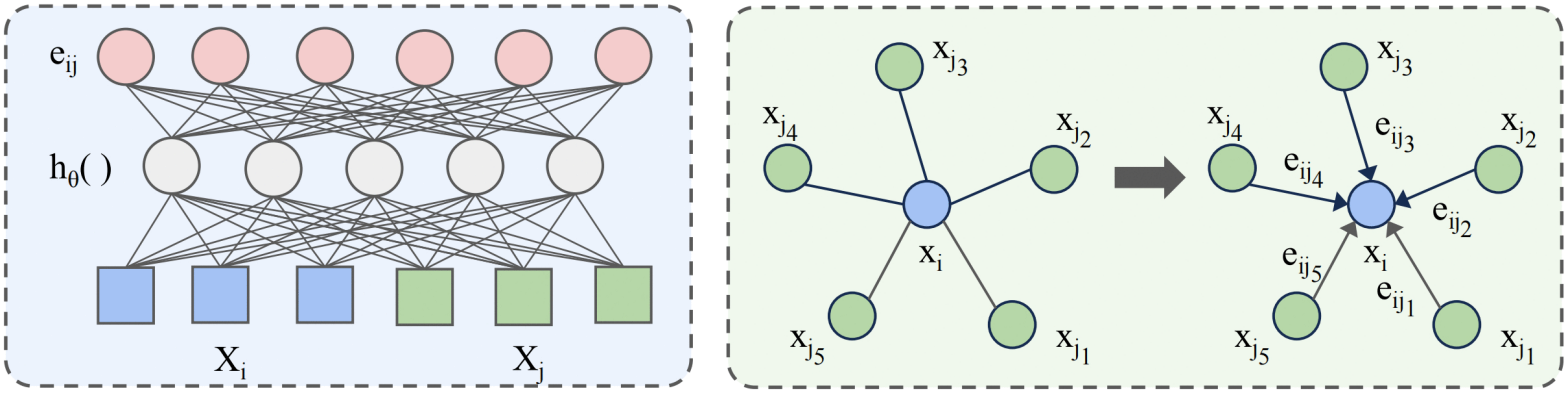
Edge feature computation and aggregation.

However, DGCNN typically implements KNN search based on CPU during the neighborhood construction stage. As the scale of point clouds increases or the training batch size grows, the neighborhood construction process can easily become a performance bottleneck in both training and inference, thereby limiting the application efficiency of the model in large-scale point cloud scenarios. To address this issue, this paper introduces a GPU-accelerated KNN search strategy in the edge feature extraction module, leveraging the advantages of parallel computing in video memory to achieve fast neighborhood construction, thereby significantly reducing the time cost of neighbor point search. This approach not only preserves the advantages of EdgeConv in local geometric modeling but also improves the efficiency of the model during training and inference, providing more efficient feature inputs for subsequent spatial attention and global feature modeling. For an input point set $X=\{{𝐱}_{i}{\}}_{i=1}^{N}$, the edge feature ${𝐞}_{ij}$ is extracted as:


$$\begin{array}{c} {𝐞}_{ij}=h_{\theta }\;\left({𝐱}_{i},\text{\textbackslash{}hspace\{0.33em}\}{𝐱}_{j}-{𝐱}_{i}\right) \end{array}$$ (1)

where $h_{\theta }$ is a shared multi-layer perceptron (MLP). Through edge feature extraction, the sensitivity to boundary features of surface defects is enhanced. The computation process is shown in Fig 2. By jointly mapping the central point features and neighborhood difference features, this module not only highlights regions with significant local geometric changes but also suppresses interference from irrelevant background points to a certain extent. For the task of wire rope surface defect segmentation, this neighborhood relationship-based feature representation method can more effectively preserve the boundary information and local morphological characteristics of defect regions, laying a foundation for subsequent modules to further model local spatial relationships and global dependencies.

### 3.2. Spatial attention module

**Fig 3:**
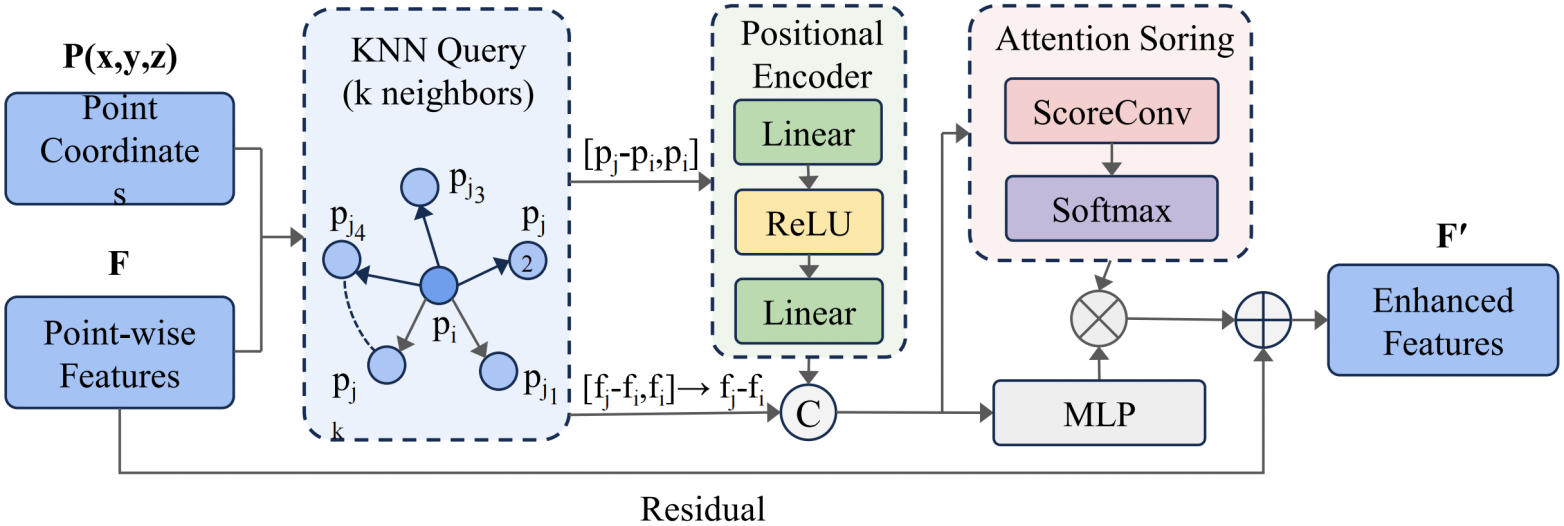
SAM module architecture.

Unlike DGCNN, which relies solely on relative differences to construct edge features, this paper further introduces a joint positional encoding that combines absolute coordinates and relative positions within the Spatial Attention Module (SAM). This enables the model to explicitly model the spatial distribution of points while maintaining local geometric sensitivity, thereby avoiding the information insufficiency caused by over-reliance on local differences during feature extraction. On this basis, SAM not only strengthens the representation of geometric relationships but also overcomes the limitation of traditional spatial attention mechanisms in point cloud processing, where weights are assigned solely based on feature similarity. Due to the sparse and unstructured nature of point clouds, feature-only attention often fails to accurately capture the spatial relationships between points. By incorporating absolute spatial position information, the proposed SAM provides explicit geometric constraints for the attention mechanism, significantly enhancing the model’s representational power and robustness in complex, non-uniform point cloud scenarios. The overall structure is shown in Fig 3.

Specifically, given the input point-wise feature representation $𝐅\in {\mathbb{R}}^{B\times 6\times N}$ and the corresponding spatial coordinates $𝐩\in {\mathbb{R}}^{B\times 3\times N}$, where *B* denotes the batch size, 6 is the number of feature channels, and *N* is the number of points, the neighborhood index $\text{idx}\in {\mathbb{N}}^{B\times N\times k}$ is first constructed using GPU-KNN. Then, the positional encoding PosEnc_ij_ is generated by concatenating the relative coordinates $\Delta {𝐩}_{ij}={𝐩}_{j}-{𝐩}_{i}$ and the absolute coordinates ${𝐩}_{i}$, which is formulated as:


$$\begin{array}{c} {\text{PosEnc}}_{ij}=\phi \;\left(\left[\Delta {𝐩}_{ij},\text{\textbackslash{}hspace\{0.17em}\}{𝐩}_{i}\right]\right) \end{array}$$ (2)

where *ϕ* denotes a two-layer fully connected network. This positional encoding explicitly models the geometric relationship between a point and its neighbors, enabling the attention distribution to better conform to the underlying spatial structure. Subsequently, the positional encoding is concatenated with the neighborhood feature difference to obtain a fused feature representation. This representation is then fed into two parallel branches for adaptive neighborhood information aggregation. The feature mapping branch uses a multi-layer perceptron (MLP) to transform the concatenated feature into a refined neighborhood feature ${𝐞}_{ij}$ representation:


$$\begin{array}{c} {𝐞}_{ij}=MLP\;\left(\left[{𝐟}_{j}-{𝐟}_{i},\text{\textbackslash{}hspace\{0.33em}PosEnc{\}}_{ij}\right]\right) \end{array}$$ (3)

where ${𝐟}_{j}-{𝐟}_{i}$ denotes the feature difference between the neighboring point *j* and the center point *i*, which is used to encode local feature variation. Subsequently, the attention scoring branch employs a two-layer convolutional network ψ (with channels progressively reduced from 2C to 1) to generate a scalar score for each neighboring point. After normalization along the neighborhood dimension using Softmax, the attention weight is obtained as:


$$\begin{array}{c} w_{ij}=\frac{exp\;\left(\psi \;\left(\left[{𝐟}_{j}-{𝐟}_{i},\text{\textbackslash{}hspace\{0.33em}PosEnc{\}}_{ij}\right]\right)\right)}{\sum_{k\in idx}exp\;\left(\psi \;\left(\left[{𝐟}_{k}-{𝐟}_{i},\text{\textbackslash{}hspace\{0.33em}PosEnc{\}}_{ik}\right]\right)\right)} \end{array}$$ (4)

where $w_{ij}$ represents the scalar attention weight assigned to neighboring point *j*, jointly determined by the geometric positional encoding and the feature difference, and satisfies $\sum w_{ij}=1$. Unlike max pooling, which aggregates all neighbors with equal importance, the scalar attention mechanism assigns continuous weights to each neighboring point through a learnable scoring function, allowing the aggregation process to adaptively distinguish relevant from irrelevant neighborhood information. Finally, the outputs of the two branches are multiplied element-wise and then summed along the neighborhood dimension in a weighted manner to achieve attention-guided neighborhood aggregation. The aggregated feature ${𝐅}^{\prime }$ is added to the original input feature 𝐅 through a residual connection ${\hat{𝐱}}_{i}$, and the output of the SAM module is given by:


$$\begin{array}{c} {𝐅}^{\prime }=𝐅+{\hat{𝐱}}_{i}=𝐅+\sum_{j\in idx}w_{ij}\text{\textbackslash{}hspace\{0.17em}\}{𝐞}_{ij} \end{array}$$ (5)

Absolute feature information has already been preserved through residual connections, so the fusion stage only needs to focus on relative changes between neighborhoods, thereby avoiding information redundancy. SAM not only compensates for the deficiency of the edge feature module in characterizing absolute spatial distribution but also provides input features with stronger geometric constraints for subsequent global dependency modeling, thus enhancing the network’s ability to recognize small-target, sparsely distributed defects.

### 3.3. Feature fusion module

**Fig 4:**
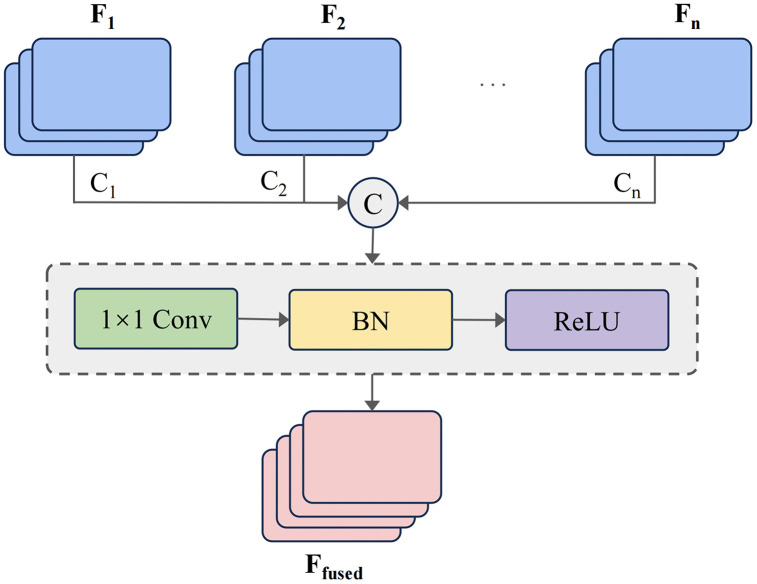
FFM module architecture.

To balance local geometric details and global context information, this paper designs a Feature Fusion Module (FFM). The core objective of the FFM is to provide input features with high information density and low computational complexity for the subsequent Transformer architecture. This module achieves feature fusion by concatenating feature maps from different channels, followed by 1 × 1 convolution, batch normalization, and ReLU activation. Its overall structure is shown in Fig 4.

Through this design, the FFM maintains computational efficiency while enabling the fused features to better adapt to the high-dimensional mapping requirements of the self-attention mechanism in the Transformer. It provides the Transformer with input features that contain sufficient geometric details and contextual associations. The output features of the FFM are denoted as:


$$\begin{array}{c} {𝐅}_{\text{fused}}=ReLU\;\left(BN\;\left({Conv}_{1\times 1}\;\left([{𝐅}_{\text{1}};{𝐅}_{\text{2}};\cdots ;{𝐅}_{n}]\right)\right)\right) \end{array}$$ (6)

Where ${𝐅}_{i}$ represents the input features of the i-th layer, ReLU is the nonlinear fusion operation, and BN is the batch normalization operation; Conv_1×1_ denotes the 1 × 1 convolution operation, and ${𝐅}_{\text{fused}}$ represents the output features after fusion. The EFEM and SAM modules at different levels extract local geometric features at different scales. Directly feeding these multi-scale features into the Transformer would lead to excessive dimensionality and high computational cost. The FFM uses 1 × 1 convolutions to perform cross-channel feature interaction and dimensionality reduction, compressing the multi-scale features into a compact representation of uniform dimensionality with almost no loss of information, thereby significantly reducing the complexity of subsequent self-attention computation.

### 3.4. Attention mechanism

**Fig 5:**
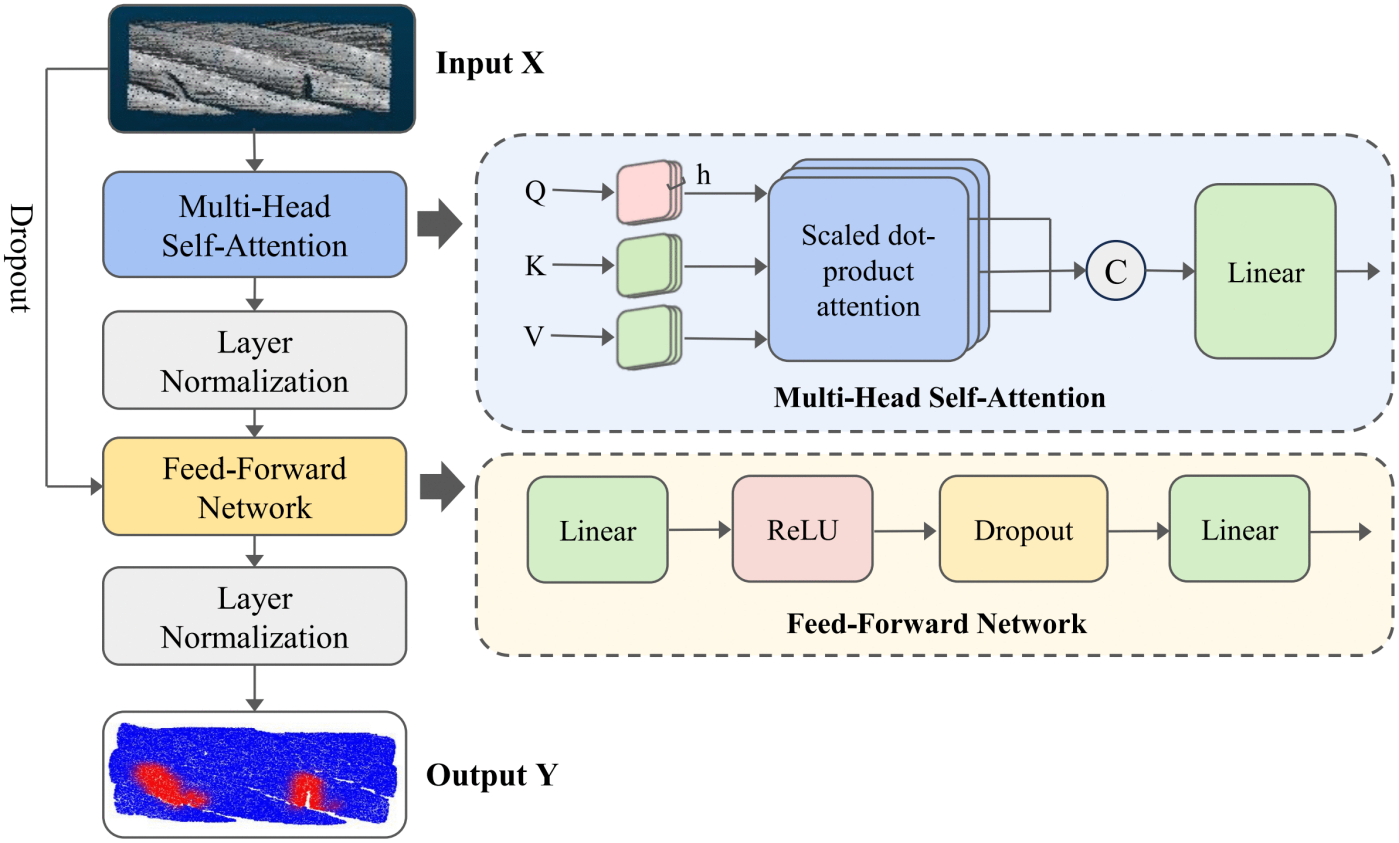
The structure of FAM mechanism.

In wire rope surface defect point clouds, defects are typically characterized by small size, sparse distribution, and complex morphology. If feature extraction relies solely on local geometric differences, small-target features can easily be overwhelmed by background points. To address this issue, this paper designs a Feature Attention Module (FAM) after the feature fusion module, which mainly consists of a multi-head self-attention unit and a feed-forward network unit, as shown in Fig 5.

In the attention heatmaps corresponding to features processed by the FAM module, red regions indicate high feature correlation, while blue regions indicate lower attention. Unlike traditional DGCNN, which relies solely on edge feature modeling within local neighborhoods, the self-attention mechanism in the FAM module can explicitly model correlations between any two points in the input sequence, thereby achieving dynamic capture of global dependencies. This design not only strengthens the model's global perception capability in complex point cloud scenes but also effectively improves segmentation accuracy and robustness for small-target defects.

Specifically, consider the input as $𝐗\in {\mathbb{R}}^{B\times N\times d_{m}}$, where *B* is the batch size, *N* is the sequence length (number of input points), and $d_{m}$ is the model dimension. The linear projection is:


$$\begin{array}{c} 𝐐=𝐗{𝐖}^{Q},\text{\textbackslash{}hspace\{1em}\}𝐊=𝐗{𝐖}^{K},\text{\textbackslash{}hspace\{1em}\}𝐕=𝐗{𝐖}^{V} \end{array}$$ (7)

The 𝐐,𝐊,𝐕are split into *h* heads according to the feature dimension, with each head having a dimension of $d_{k}=d_{m}/h$. The processing per head is:


$$\begin{array}{c} 𝐐,𝐊,𝐕\in {\mathbb{R}}^{B\times N\times d_{m}}\text{\textbackslash{}hspace\{0.33em}\}\to \text{\textbackslash{}hspace\{0.33em}\}𝐐,𝐊,𝐕\in {\mathbb{R}}^{B\times h\times N\times d_{k}} \end{array}$$ (8)

The attention scores are calculated as:


$$\begin{array}{c} Attention\left(𝐐,𝐊,𝐕\right)=softmax\;\left(\frac{𝐐{𝐊}^{⊤}}{\sqrt{d_{k}}}\right)𝐕 \end{array}$$ (9)

The resulting attention distribution measures the correlation between points and aggregates global contextual information accordingly. The multi-head mechanism enables the model to learn diverse geometric relationship patterns in parallel across different subspaces, allowing small-scale defect regions to be amplified and highlighted within the global context. Finally, the outputs from all heads are concatenated and projected back to the original dimension, serving as globally enhanced features that are input to the FFN, which further introduces nonlinear transformation and feature remapping, thereby achieving explicit enhancement of small-target defects.

## 4. Experiments and results

### 4.1. Experimental dataset

#### 4.1.1. Construction of the experimental workbench.

**Fig 6:**
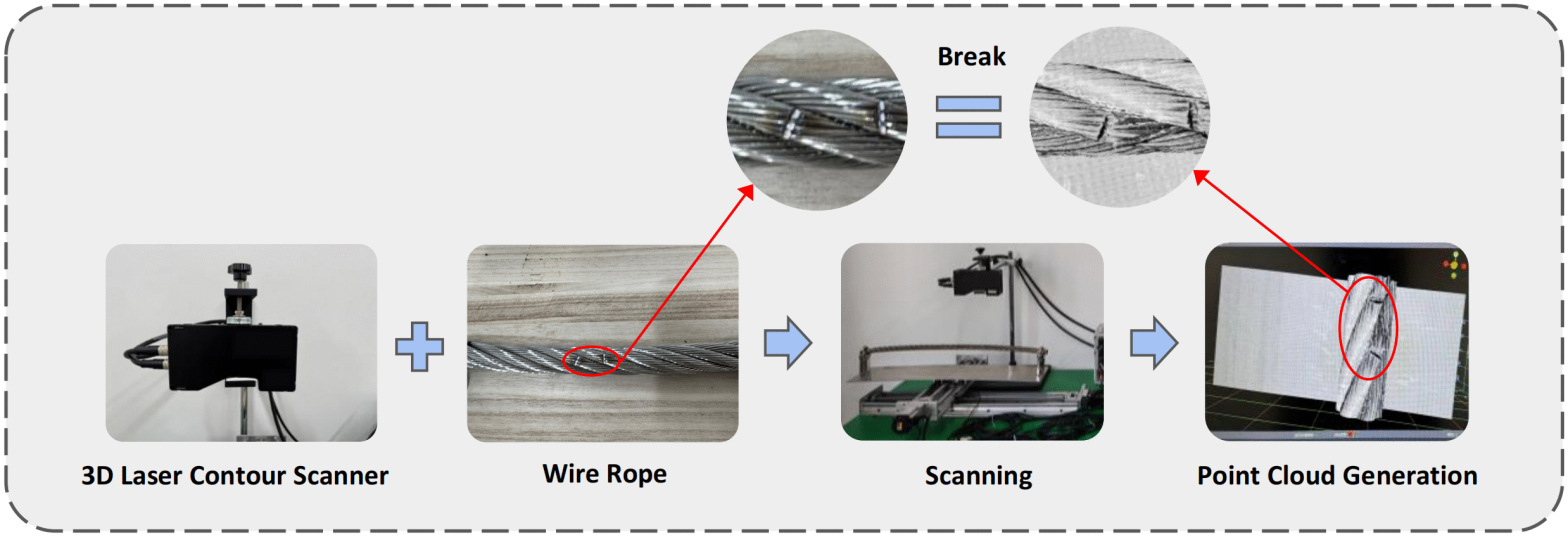
Experimental workbench. All photographs were taken by the authors.

This study establishes an indoor laboratory scanning point cloud data acquisition platform, which mainly consists of a 3D laser profiler, an XY-axis displacement mechanism, a motion controller, a workbench, and a computer. As shown in Fig 6, the 3D laser profile scanner is used to scan the surface of the wire rope to obtain point cloud data. During the entire scanning process, the calibration and configuration of the sensor system are strictly controlled to ensure the accuracy and reliability of the data.

The core acquisition device of the platform is a 3D laser profile sensor, model LVM L2240 (manufactured by Tianjin Yike Automation Co., Ltd.), with a laser wavelength of 405 nm and laser classes 2 and 3R. This sensor precisely scans and processes object contours and dimensions using an ultra-high-speed processor, and can generate and output high-precision point cloud data. It is unaffected by the color or gloss of the workpiece and is suitable for measuring various materials such as metals, plastics, and glass.

The sensor achieves high-precision 3D inspection based on the principle of triangulation: its built-in line laser projects laser light onto the surface of the wire rope, and an internal camera captures the reflected light signal from a specific angle. Since the distance from the sensor to each point on the wire rope surface varies, the position of the reflected light on the camera’s imaging plane changes accordingly, forming a measurement triangle among the laser emitter, the camera, and the target object. Using the pre-calibrated sensor parameters provided by the manufacturer, the height of each point on the laser profile can be calculated, enabling high-precision monitoring of subtle deformations and morphological features of the wire rope. Specifically, the sensor has an X-direction resolution of 8.6–28.0 μm and a Z-direction resolution of 42.0–77.2 μm, meeting the requirements for monitoring subtle deformations of the wire rope.

#### 4.1.2. Data preprocessing.

Currently, there is no publicly available dataset for the segmentation and recognition of surface defects on wire ropes based on point cloud data. Therefore, this study constructs its own wire rope defect point cloud dataset, named WireRope3D, which covers three common types of surface damage: wear, wire protrusion (incomplete fracture), and broken wire (complete wire fracture). These three defect types were selected due to their high frequency of occurrence in industrial applications, making them typical and representative. The wire rope used in the experiment is a 304 stainless steel wire rope with a diameter of 22 mm. During the acquisition process, the wire rope was fixed on a workbench, and the XY-axis moving mechanism was controlled to move it along the Y-axis. Meanwhile, a laser profile sensor was used to scan its surface and output point cloud data. To comprehensively cover the spatial characteristics of defects from different viewing angles, point cloud acquisition was performed on each defect region under various rotation conditions, thereby ensuring the diversity and completeness of the dataset.

Since the 3D laser profile sensor is susceptible to equipment noise, local reflectance variations, and sampling discreteness when scanning the wire rope surface, the initially acquired point cloud data inevitably contain issues such as outliers, local burrs, and surface fluctuations. If left unprocessed, these noises not only weaken the geometric differences between defect regions and normal regions but may also interfere with subsequent manual annotation and model training, leading to inaccurate segmentation boundaries and unstable feature learning. Therefore, before the data enters the annotation and training stages, this paper first performs preprocessing on the raw point cloud to improve point cloud quality and enhance data consistency.

**Fig 7:**
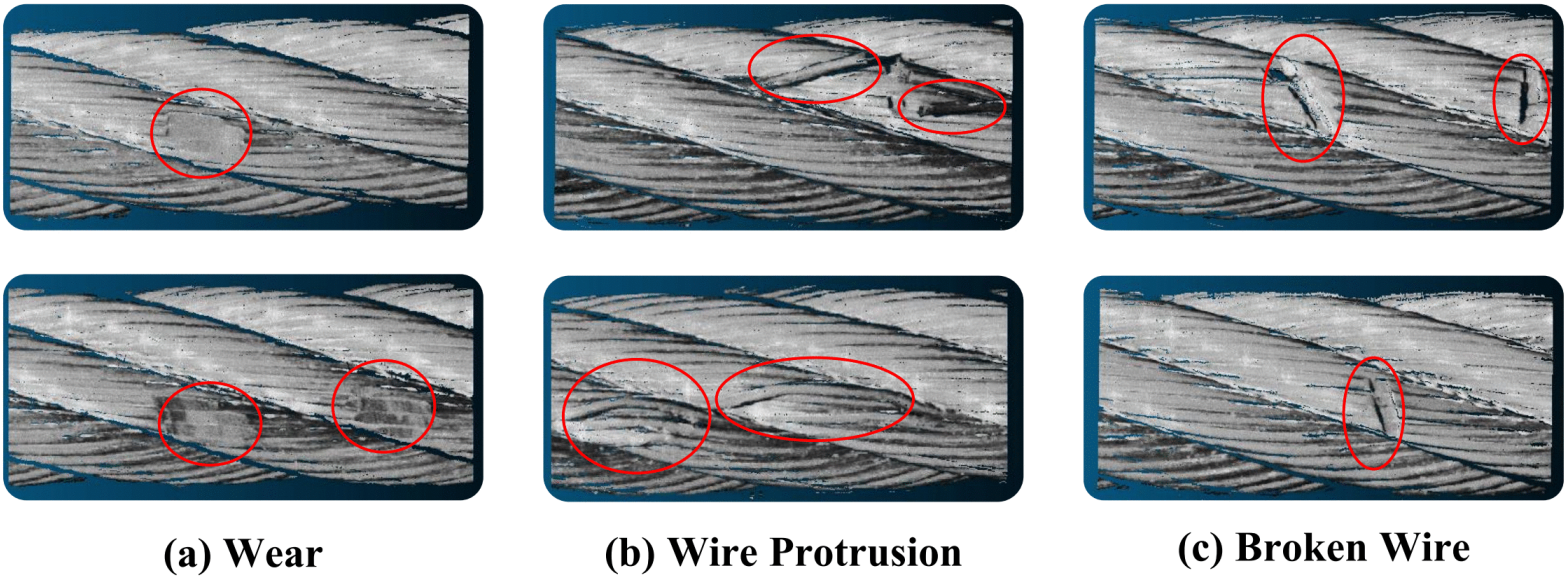
Point cloud examples of typical wire rope defects.

Specifically, the preprocessing process mainly consists of two steps: radius filtering and Gaussian filtering. The role of radius filtering is to remove isolated points and outliers with insufficient points within a local neighborhood, thereby reducing the damage of random noise to the overall geometric structure. For targets such as wire ropes with continuous strand textures, outliers typically appear as anomalous scattered points detached from the main surface. If retained directly, they can easily be misidentified by the model as small-scale defects. Through radius filtering, the continuous surface structure of the wire rope main body can be effectively preserved, improving the overall usability of the point cloud. On this basis, Gaussian filtering is further applied to smooth the point cloud surface, reducing high-frequency fluctuations caused by scanning jitter and uneven local reflectance, making the point cloud smoother while maintaining the main geometric contours and defect morphologies. After the above processing, the typical defect point clouds on the wire rope surface become clearer. As shown in Fig 7, the preprocessed point clouds can better retain the main spatial characteristics of defects such as wear, wire protrusions, and broken wires, providing a more reliable data foundation for subsequent fine segmentation.

**Fig 8:**
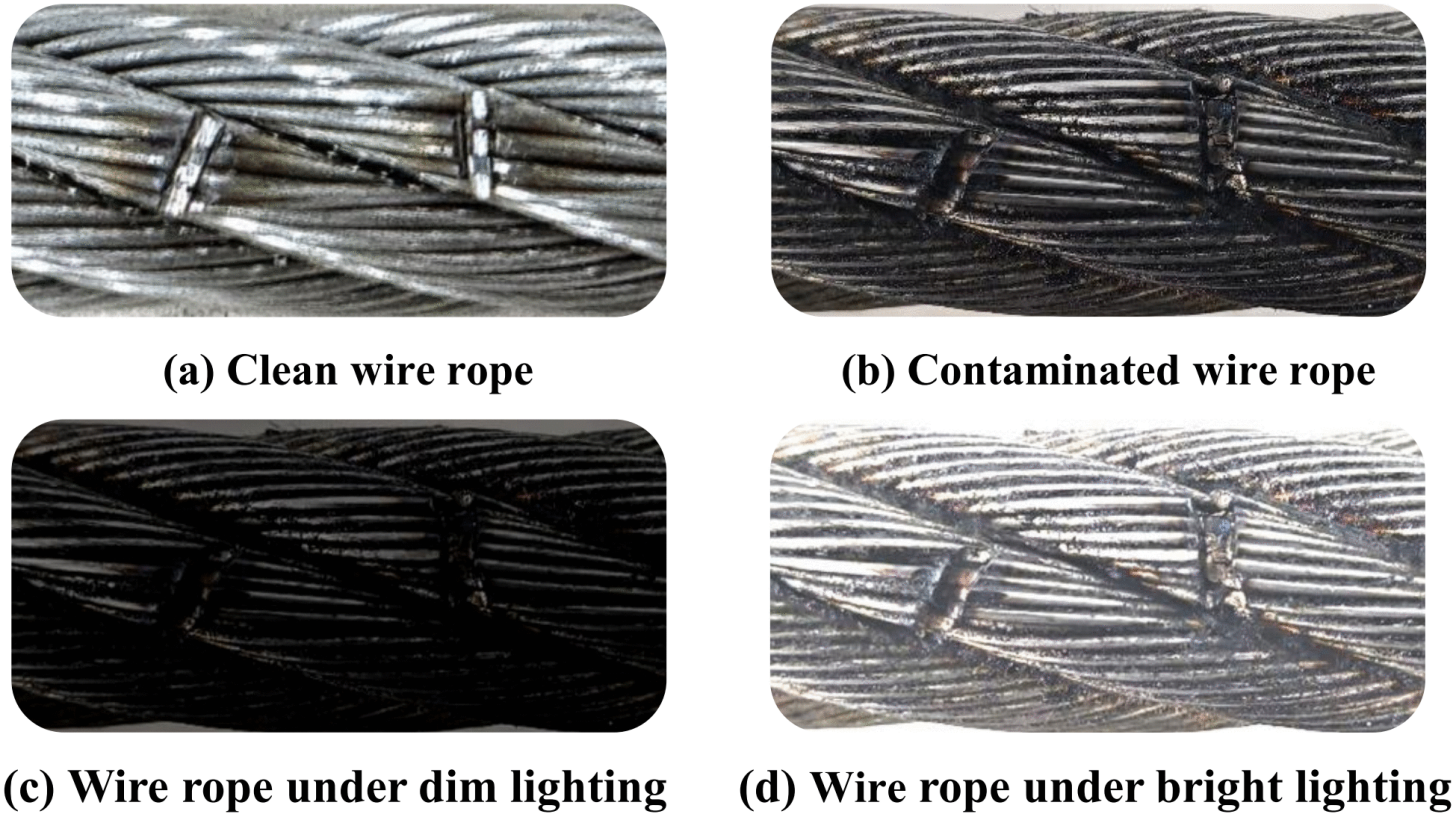
Appearance of wire ropes under different surface and lighting conditions.

To visually illustrate variations in the appearance of wire ropes under different surface and illumination conditions, Fig 8 presents four representative scenarios: a clean surface, oil and coal-dust contamination, low illumination, and high illumination. These scenarios simulate common non-ideal conditions encountered in mining environments. Because the 3D laser profile sensor acquires surface geometric information based on active laser triangulation, point cloud acquisition is largely independent of external illumination. Consequently, variations in ambient illumination within a normal range have no noticeable effect on wire rope point cloud acquisition. To mitigate point cloud noise caused by oil and coal dust, radius-based filtering is applied during preprocessing to remove isolated points and outliers, followed by Gaussian filtering to suppress local high-frequency fluctuations. This procedure improves point cloud quality while preserving defect boundaries and geometric morphology as much as possible.

**Fig 9:**
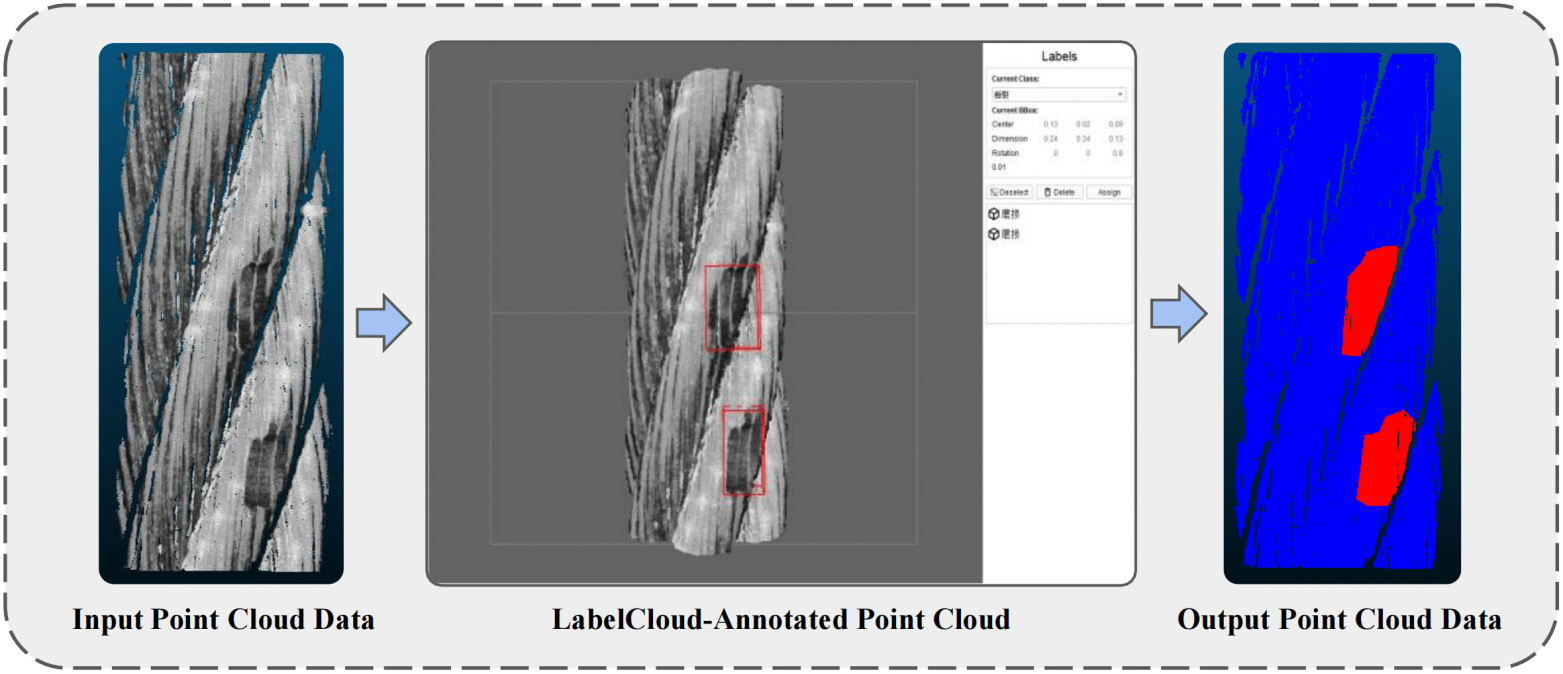
Semantic segmentation annotation of point cloud data.

To meet the requirements of experimental research, the collected wire rope point clouds were further manually annotated. After completing point cloud denoising and smoothing, this paper performed manual annotation on the collected wire rope point clouds. As shown in Fig 9, the LabelCloud tool was used to perform semantic segmentation annotation on the point cloud data, where blue represents the original point cloud and red represents points assigned with defect labels. This annotation method can accurately distinguish defect regions from normal regions at the point level, thereby preserving the spatial extent and boundary information of defects more completely. This provides solid data support for subsequent defect segmentation experiments and effectively improves the accuracy of point cloud segmentation.

To ensure annotation reliability, the point cloud samples were annotated by two researchers familiar with wire rope defect morphology and point cloud processing. Before annotation, unified criteria for identifying each defect type were established, and boundary delineation rules were standardized using preliminary annotation samples. After annotation, all samples were reviewed, with particular attention paid to ambiguous boundaries, class confusion, and missed regions. Samples with disputed annotations were discussed and revised accordingly, resulting in the final semantic labels used for model training and testing.

#### 4.1.3. Dataset construction.

The WireRope3D dataset constructed in this study contains three typical types of wire rope surface defects: wear, wire protrusion, and broken wire. Wire protrusion refers to the local outward displacement or incomplete fracture of a wire, whereas broken wire refers to its complete fracture. The original point cloud samples were acquired using a 3D laser profile sensor, yielding a total of 696 original scans. Considering that wire rope surfaces in coal mine environments may be affected by dust deposition, oil contamination, and variations in surface reflectivity, the collected data include point cloud samples acquired under several non-ideal surface conditions, thereby enhancing the dataset’s ability to represent complex operating conditions.

The dataset was partitioned at the level of original scans and physical defect locations to ensure that samples originating from the same source were not distributed across different subsets. Subsequently, data augmentation was performed using random rotation and scaling, with the scaling factor randomly selected within the range of 0.8–1.2. Through augmentation, the 696 original scans were expanded to 2,916 point cloud samples, comprising 2,058 training samples, 427 validation samples, and 431 test samples. The distribution of samples across the three defect categories is presented in Table 1.

**Table 1:** Sample count by defect category.

Defect Category	Original samples	Defect locations	Augmented samples	Training Set	Validation Set	Test Set
Wear	234	303	982	694	145	143
Wire Protrusion	229	298	961	678	140	143
Broken Wire	233	318	973	686	142	145
Total	696	919	2916	2058	427	431

Because sample counts do not adequately represent class distributions in point-level semantic segmentation, Table 2 reports the point-level statistics for the training, validation, and test sets. The defect-point proportion was calculated as the combined number of wear, wire protrusion, and broken wire points divided by the total number of points. Defect points account for only 3.6148% of WireRope3D, confirming their highly sparse distribution. Their proportions in the training, validation, and test sets are 3.5915%, 3.6496%, and 3.6911%, respectively, indicating consistent point-level class distributions across the three subsets.

**Table 2:** Point-level class distribution of the WireRope3D dataset.

Dataset	Background points	Wear points	Wire protrusion points	Broken wire points	Total points	Proportion of defect points
Training set	32,507,267	448,689	561,207	201,109	33,718,272	3.5915%
Validation set	6,740,641	94,129	119,162	42,036	6,995,968	3.6496%
Test set	6,800,859	94,365	126,371	39,909	7,061,504	3.6911%
Total	46,048,767	637,183	806,740	283,054	47,775,744	3.6148%

**Fig 10:**
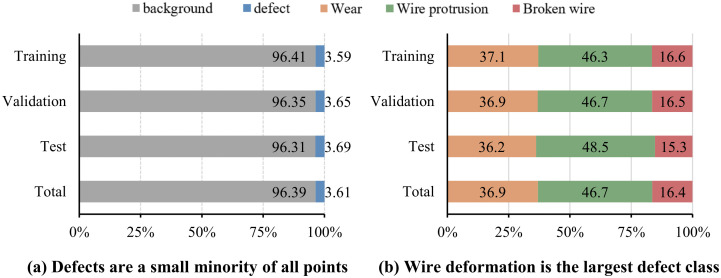
Class Imbalance and Defect Composition in WireRope3D.

To more intuitively illustrate the imbalance between background and defect points, as well as the composition of different defect classes within the defect points, a visualization of the point-level class distribution is presented in Fig 10. As shown in Fig 10, defect points account for only a small proportion of all points in each dataset subset, with relatively minor variations among the subsets. Moreover, among the defect points, wire protrusion accounts for the largest proportion, followed by wear, whereas broken wire accounts for the smallest proportion. This distribution further demonstrates the differences among defect classes at the point level.

### 4.2. Experimental environment

The experimental environment is based on the Ubuntu 20.04 operating system, Intel(R) Xeon(R) Platinum 8481C CPU, 90 GB of memory, and an RTX 4090 graphics card. The deep learning framework is PyTorch 2.0.0 with Python 3.8. We use the SGD optimizer with a momentum of 0.9. The initial learning rate is 0.1, which is reduced to 0.0003 using cosine annealing [33]. This setting ensures that the network achieves good segmentation accuracy after convergence. The input to the segmentation network consists of 2,048 uniformly sampled points, the number of neighboring points k is set to 20, and the number of training epochs is 100. The training batch size is 16, and the test batch size is 8.

### 4.3. Evaluation metrics

To verify the performance of the proposed network for the wire rope point cloud defect segmentation task, point cloud part segmentation experiments were conducted on our dataset. The mean Intersection over Union (mIoU), which represents the ratio of the intersection to the union of the ground truth and predicted values for all classes, and the mean accuracy (mAcc) are used as evaluation metrics for the proposed method, as shown in Equations (10) and (11).


$$\begin{array}{c} \text{mAcc}=\frac{\text{1}}{C}\sum_{c=1}^{C}\frac{T_{c}}{T_{c}+N_{c}} \end{array}$$ (10)

$$\begin{array}{c} \text{mIoU}=\frac{\text{1}}{C}\sum_{c=1}^{C}\frac{T_{c}}{T_{c}+F_{c}+N_{c}} \end{array}$$ (11)

Where T_c_ represents the number of points correctly predicted as class c; N_c_ represents the number of points whose ground truth is class c but are predicted as other classes; and F_c_ represents the number of points whose ground truth is not class c but are predicted as class c.

### 4.4. Experimental results

#### 4.4.1. Ablation experiments.

To further investigate the importance of different modules in the network architecture, ablation experiments were conducted on our self-constructed dataset. Experiments were performed sequentially on the Spatial Attention Module (SAM), Feature Fusion Module (FFM), and Feature Attention Module (FAM) to study the impact of each module on the test results. The experimental results are shown in Table 3.

**Table 3:** Ablation experiments of different modules.

Number	Baseline	EFEM	SAM	FFM	FAM	Per-class IoU(%)			Defect mIoU(%)	mIoU(%)	mAcc(%)	Parameters (M)	FLOPs (G)	Inference Time (ms)	Memory consumption (GB)
						Wear	Wire Protrusion	Broken Wire							
1	√					75.87	71.19	57.65	68.24	75.91	82.54	1.83	2.58	28.76	1.87
2	√	√				76.58	70.12	58.38	68.36	76.03	83.09	1.86	2.72	29.24	1.82
3	√	√	√			77.25	71.65	61.37	70.09	77.36	83.32	1.91	2.94	30.05	1.76
4	√	√	√	√		77.43	70.65	61.81	69.96	77.28	83.24	1.94	3.02	30.38	1.69
5	√	√	√	√	√	**79.71**	**74.04**	**68.37**	**74.04**	**80.32**	**85.63**	2.02	3.29	31.41	1.62

**Fig 11:**
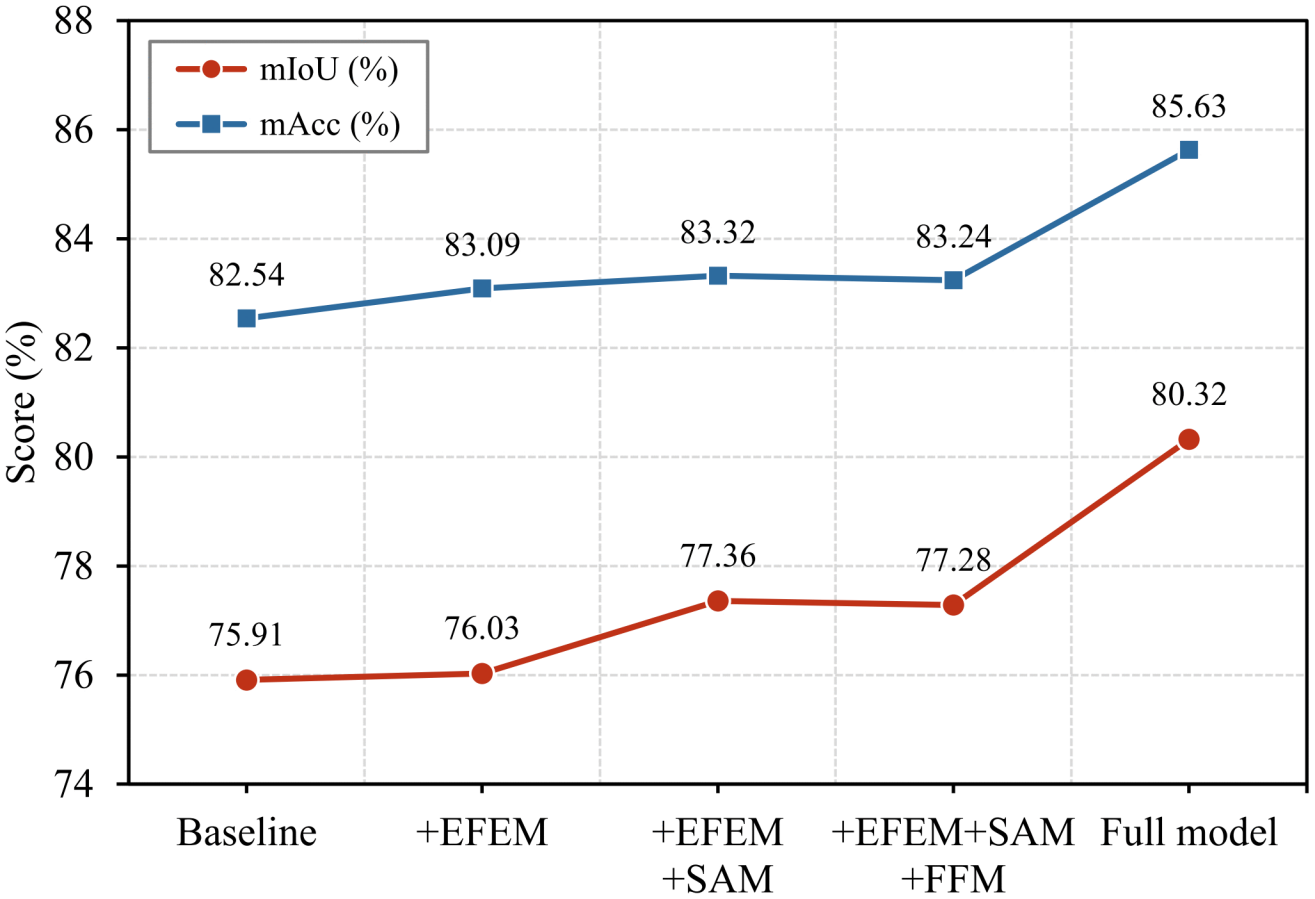
Performance variation curve with the gradual introduction of each module.

Based on the quantitative comparisons in Table 3, the variation curves of the principal evaluation metrics, including Overall mIoU, Defect mIoU, and mAcc, were further plotted to more intuitively illustrate the changes in segmentation performance as the functional modules were progressively introduced, as shown in Fig 11. Compared with the baseline number 1, adding EFEM in number 2 improves both Overall mIoU and Defect mIoU by 0.12 percentage points, indicating that neighborhood edge features supplement local geometric modeling. Incorporating SAM further increases Overall mIoU from 76.03% to 77.36% and Defect mIoU from 68.36% to 70.09%, with a 2.99-point improvement in broken wire IoU, demonstrating enhanced sensitivity to local geometric anomalies and defect boundaries.

After adding FFM, number 4 remains comparable to number 3, with only 0.08- and 0.13-point decreases in Overall mIoU and Defect mIoU, respectively. This indicates that FFM provides compact multi-level feature fusion without substantially compromising segmentation performance. The subsequent introduction of FAM increases Overall mIoU, Defect mIoU, and mAcc by 3.04, 4.08, and 2.39 percentage points, respectively, including a 6.56-point improvement in broken wire IoU. Overall, the complete number 5 model outperforms number 1 by 4.41 points in Overall mIoU, 5.80 points in Defect mIoU, and 3.09 points in mAcc, confirming the complementarity of local geometric and global contextual modeling.

Model complexity increases moderately as the modules are progressively introduced. From number 1 to number 5, the parameter count increases from 1.83 M to 2.02 M, FLOPs from 2.58 G to 3.29 G, and inference time from 28.76 to 31.41 ms. Although this corresponds to increases of 10.38% in parameters and 9.21% in inference time, Overall mIoU and Defect mIoU improve by 4.41 and 5.80 percentage points, respectively, indicating a favorable accuracy–efficiency trade-off.

Notably, peak GPU memory consumption decreases from 1.87 to 1.62 GB (13.37%), possibly because feature selection, compression, and fusion reduce redundant intermediate activations. With FFM, the parameter count increases only from 1.91 M to 1.94 M, FLOPs from 2.94 G to 3.02 G, and inference time by 0.33 ms, while GPU memory consumption decreases from 1.76 to 1.69 GB. Thus, FFM primarily provides compact multi-level feature fusion and a more efficient input representation for subsequent global feature recalibration by FAM. Although FAM introduces additional computational overhead, it improves Defect mIoU by 4.08 percentage points, demonstrating a substantial performance benefit.

To isolate the contributions of relative positions and absolute coordinates in SAM, four encoding strategies were evaluated while keeping all other network configurations and training conditions unchanged: no positional encoding, relative positions only, absolute coordinates only, and their joint encoding. The results are presented in Table 4.

**Table 4:** Ablation Study of Different Positional Encoding Strategies.

Number	Relative Position	Absolute Coordinates	Per-class IoU(%)			Defect mIoU(%)	mIoU(%)	mAcc(%)
			Wear	Wire Protrusion	Broken Wire			
1			78.12	72.58	64.71	71.80	78.59	84.41
2	√		79.13	73.55	67.21	73.30	79.74	85.15
3		√	78.64	72.96	66.28	72.63	79.23	84.86
4	√	√	79.71	74.04	68.37	74.04	80.32	85.63

As shown in Table 4, the model without positional encoding achieves an Overall mIoU of 78.59% and a Defect mIoU of 71.80%. Relative positional encoding increases these metrics to 79.74% and 73.30%, respectively, confirming that coordinate differences between central and neighboring points capture local geometric variations. Absolute coordinate encoding achieves 79.23% and 72.63%, indicating its ability to represent spatial constraints within the helical rope structure. Combining both encodings yields the best results, with an Overall mIoU of 80.32%, a Defect mIoU of 74.04%, and an mAcc of 85.63%. These values exceed those obtained without positional encoding by 1.73, 2.24, and 1.22 percentage points, respectively, while broken wire IoU improves by 3.66 points. The results demonstrate that relative positions and absolute coordinates provide complementary local geometric and global spatial information.

**Fig 12:**
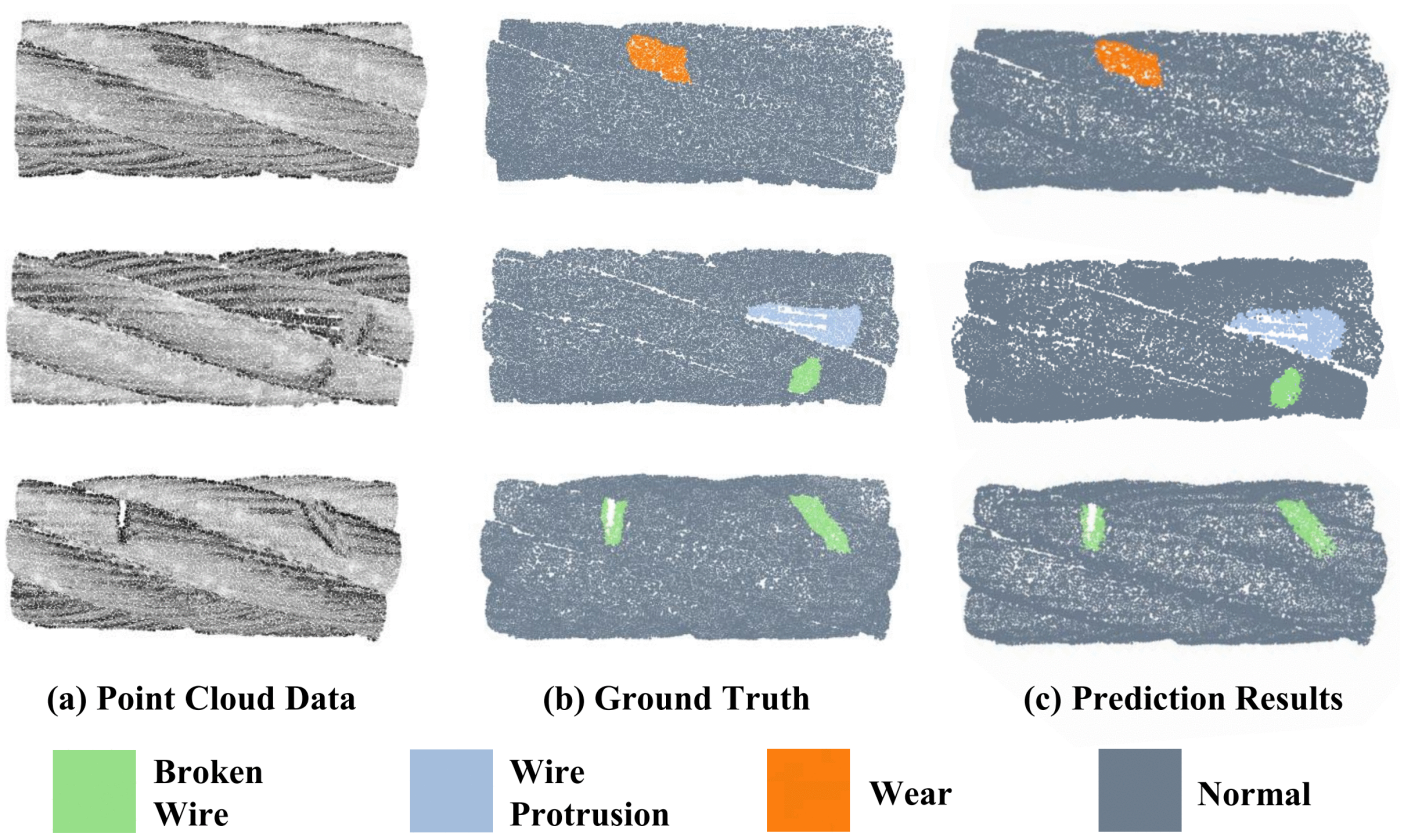
Examples of point cloud segmentation on the WireRope3D dataset.

To more intuitively demonstrate the point cloud segmentation effect, Fig 12 presents some visualization results of part segmentation.

#### 4.4.2. Comparative experiments.

To verify the performance of the proposed network for the task of wire rope point cloud defect segmentation, point cloud semantic segmentation experiments are conducted on the WireRope3D dataset. Four representative methods were selected for comparison: PointNet++, DGCNN, Point Transformer, and Point S-GS Former. The results are shown in Table 5.

**Table 5:** WireRope3D Segmentation results.

Method	Per-class IoU(%)			Defect mIoU(%)	mIoU(%)	mAcc(%)
	Wear	Wire Protrusion	Broken Wire			
PointNet++ [18]	69.10	65.20	49.90	61.40	70.64	**87.04**
DGCNN [20]	75.87	71.19	57.65	68.24	75.91	82.54
Point Transformer [22]	76.52	**77.96**	63.79	72.76	78.64	84.72
Point S-GS Former [16]	**80.26**	76.52	64.89	73.89	80.18	85.21
Ours	79.71	74.04	**68.37**	**74.04**	**80.32**	85.63

**Fig 13:**
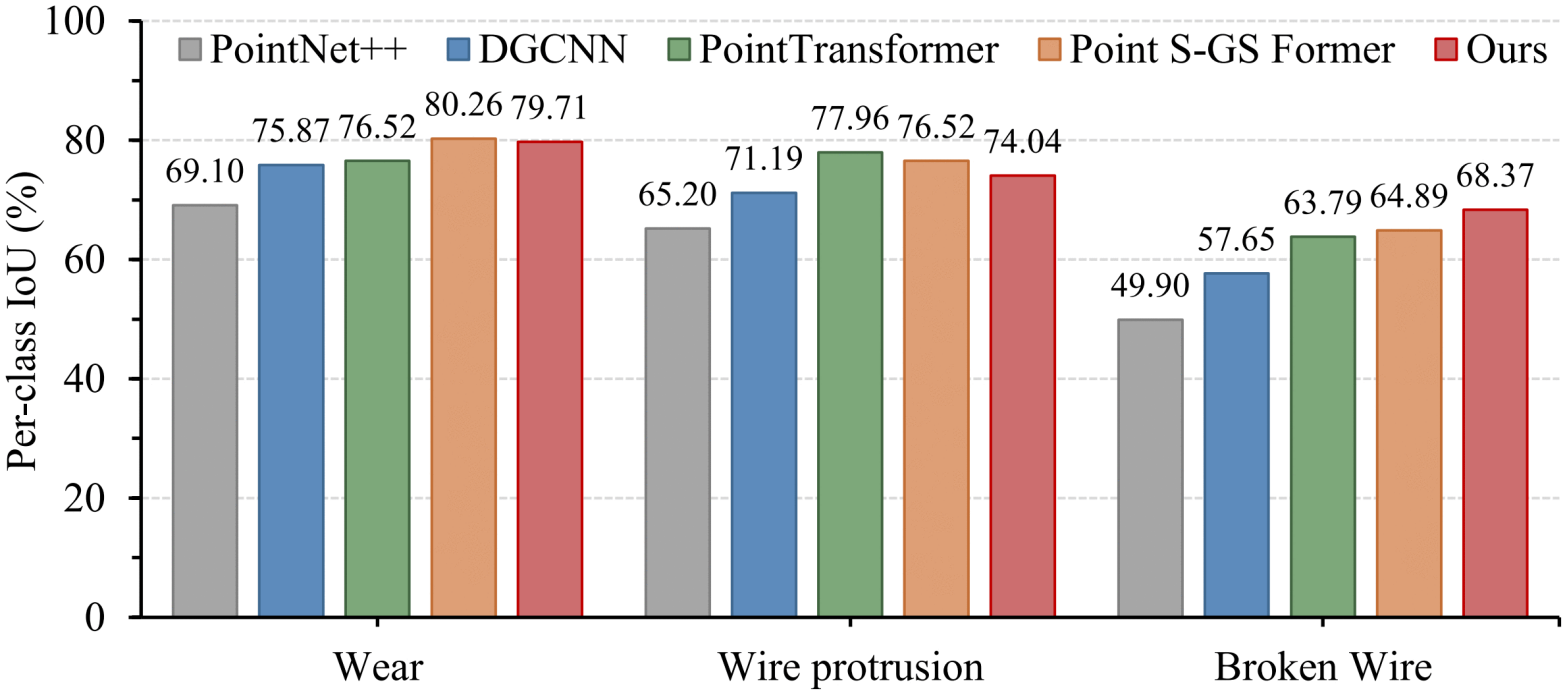
Bar chart of segmentation results on WireRope3D.

As shown in Table 5 and Fig 13, the proposed method achieves an Overall mIoU of 80.32% on WireRope3D, outperforming PointNet++, DGCNN, and Point Transformer by 9.68, 4.41, and 1.68 percentage points, respectively. Its Defect mIoU reaches 74.04%, exceeding PointNet++ and DGCNN by 12.64 and 5.80 points. Compared with the strongest competing method, Point S-GS Former, the proposed method provides modest gains of 0.14 points in Overall mIoU and 0.15 points in Defect mIoU.

Notably, the proposed method achieves the highest class-specific IoU for broken wire (68.37%), outperforming DGCNN by 10.72 percentage points. This result suggests that the collaboration between spatial attention and self-attention improves the representation of small, morphologically complex defects. Point S-GS Former performs best on wear (80.26%), possibly because its local spatial and multi-scale geometric modeling is well suited to the broad, continuous, and gradually deformed regions characteristic of wear. Point Transformer achieves the highest IoU for wire protrusion (77.96%), likely benefiting from attention-based modeling of its relatively regular and prominent geometric structures.

**Fig 14:**
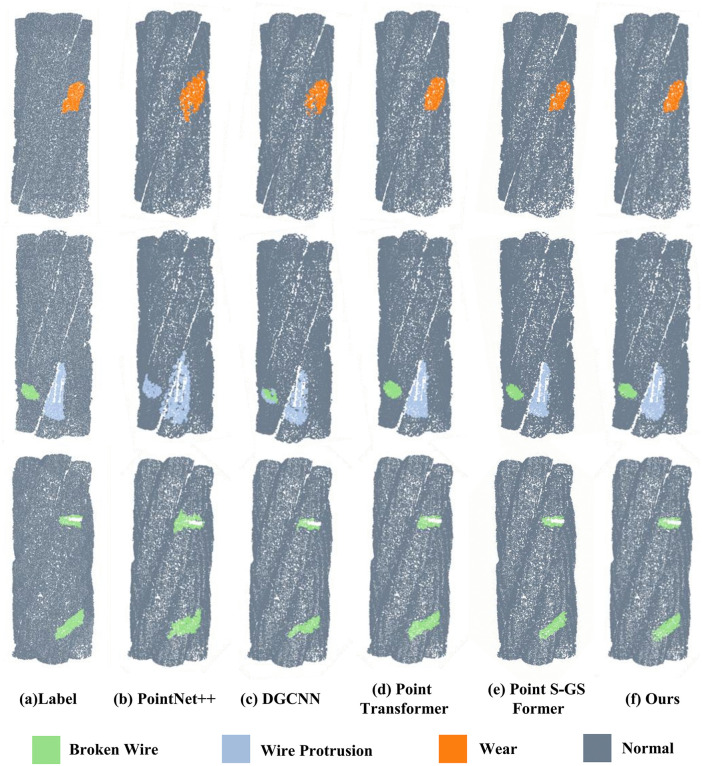
Segmentation results of different models.

Further analysis of the performance characteristics of the different methods shows that PointNet++ achieves the highest mAcc (87.04%) but a relatively low Overall mIoU (70.64%). Because mAcc is the macro-average of per-class recall, whereas IoU penalizes both false positives and false negatives, this discrepancy suggests that its high recall may be accompanied by more false-positive predictions and limited overlap with the ground truth. Therefore, mAcc alone is insufficient to assess defect-boundary segmentation quality. In contrast, the proposed method achieves higher Overall mIoU and Defect mIoU, indicating more balanced region-level segmentation. Qualitative results are presented in Fig 14.

#### 4.4.3. Model complexity analysis.

**Fig 15:**
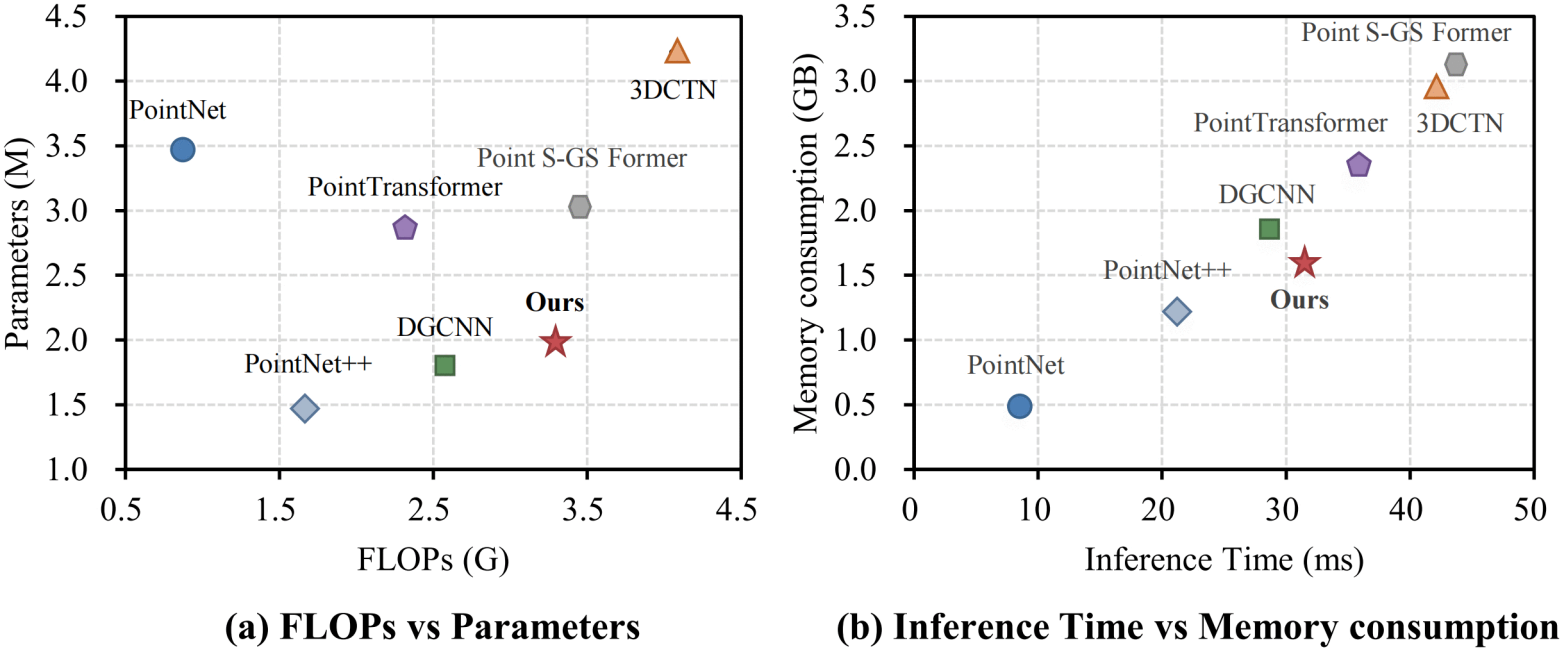
Comparison of model complexity and runtime overhead across different methods.

While ensuring segmentation accuracy, the parameter scale and computational complexity of a model are also important indicators for evaluating its practicality. To this end, this paper compares the proposed method with several representative baseline models in terms of the number of parameters, floating-point operations (FLOPs), inference time, and memory consumption, as shown in Table 6. The corresponding visual comparison is shown in Fig 15.

**Table 6:** Comparison of parameter count and computational efficiency of different models.

Method	Parameters (M)	FLOPs (G)	Inference Time (ms)	Memory consumption (GB)
PointNet [17]	3.47	0.87	8.23	0.51
PointNet++ [18]	1.48	1.69	21.51	1.24
DGCNN [20]	1.83	2.58	28.76	1.87
3DCTN [23]	4.22	4.06	42.32	2.96
Point Transformer [22]	2.88	2.32	35.60	2.34
Point S-GS Former [16]	3.04	3.47	43.50	3.12
Ours	2.02	3.29	31.41	1.62

As can be seen from Table 6 and Fig 15, the proposed method has significantly fewer parameters than PointNet, 3DCTN, Point Transformer, and Point S-GS Former and is only slightly higher than PointNet++ and DGCNN. Specifically, the proposed method has 2.02 M parameters, which is 1.02 M fewer than Point S-GS Former, indicating that although modules such as spatial attention, feature fusion, and feature attention are introduced, the overall structure remains relatively compact. In terms of computational complexity, the FLOPs of the proposed method are higher than those of PointNet, PointNet++, DGCNN, and Point Transformer, but lower than those of 3DCTN and Point S-GS Former, demonstrating that the introduction of local spatial attention and global feature attention, while increasing computational cost, effectively enhances the representation capability for complex defect point clouds.

In terms of inference efficiency, the proposed method achieves an inference time of 31.4 ms, which is faster than 3DCTN (42.3 ms) and Point Transformer (35.6 ms), and Point S-GS Former (43.50 ms), and comparable to DGCNN (28.7 ms). Although slower than the structurally simpler PointNet (8.2 ms) and PointNet++ (21.5 ms), the proposed method strikes a favorable balance between accuracy and efficiency. In particular, compared with Point S-GS Former, the proposed method reduces the inference time by 12.09 ms, indicating that the proposed modules do not introduce a severe computational bottleneck and retain good potential for real-time processing.

Regarding memory consumption, the proposed method requires only 1.62 GB, which is substantially lower than 3DCTN (2.96 GB) and Point Transformer (2.34 GB), and Point S-GS Former (3.12 GB), lower than DGCNN (1.87 GB), and slightly higher than PointNet (0.51 GB) and PointNet++ (1.24 GB). Compared with Point S-GS Former, the memory consumption is reduced by 1.50 GB. This demonstrates that while maintaining strong feature representation capability, the model still maintains a low storage overhead, making it more conducive to deployment on resource-constrained platforms such as embedded devices and mobile terminals.

From Table 6, it can be seen that the proposed method achieves an mIoU of 80.32% on the WireRope3D dataset, which is 4.41 and 1.68 percentage points higher than DGCNN and Point Transformer, respectively, and also outperforms Point S-GS Former, indicating that the additional computational cost is effectively converted into improved segmentation performance. In particular, the proposed method achieves the highest IoU for the broken wire class, demonstrating that the design combining GCN and Transformer can better balance local geometric details and global context information. Overall, the proposed method achieves a good trade-off among parameter scale, computational cost, and segmentation accuracy, and possesses certain potential for engineering applications.

#### 4.4.4. GPU-KNN Efficiency Analysis.

To independently evaluate the efficiency of GPU-KNN in neighborhood graph construction, CPU-KNN and GPU-KNN were compared under identical network architectures, input sizes, neighborhood sizes, batch sizes, model parameters, and training protocols. Both implementations used the same Euclidean-distance criterion and therefore produced identical or highly consistent neighborhoods. The experiment focused on neighborhood construction time, end-to-end inference time, and training efficiency, rather than treating GPU-KNN as an architectural component for improving segmentation accuracy. The results are presented in Table 7.

**Table 7:** Efficiency Comparison Between CPU-KNN and GPU-KNN Strategies.

KNN Method	Neighbors (k)	Train/Test Batch Size	KNN Search Time (ms)	Inference Time (ms)	Training Time (s)	Memory consumption (GB)	mIoU (%)
CPU-KNN	20	16/8	3.14	42.29	151	1.55	80.30
GPU-KNN	20	16/8	0.42	31.41	114	1.62	80.32

As shown in Table 7, GPU-KNN reduces the neighborhood construction time per sample from 3.14 to 0.42 ms (86.62%), the end-to-end inference time from 42.29 to 31.41 ms (25.73%), and the training time per epoch from 151 to 114 s (24.50%). These results confirm that neighborhood graph construction constitutes a substantial portion of the computational overhead in point cloud networks and that GPU-parallel neighbor search improves both training and inference efficiency. GPU memory consumption increases slightly from 1.55 to 1.62 GB, indicating that the acceleration requires only 0.07 GB of additional memory. Meanwhile, CPU-KNN and GPU-KNN achieve nearly identical mIoU values of 80.30% and 80.32%, respectively. Therefore, GPU-KNN primarily improves computational efficiency rather than feature representation or segmentation accuracy.

#### 4.4.5. Hyperparameter Sensitivity Analysis.

The neighborhood size k determines the coverage of local neighborhoods and directly affects the receptive field, defect-boundary perception, and computational cost of local geometric feature extraction. Because both EFEM and SAM rely on KNN-based neighborhood construction, we evaluated k∈{10,15,20,25,30} while keeping all other network configurations and training conditions unchanged. The results are presented in Table 8.

**Table 8:** Effect of Neighborhood Size k on Segmentation Performance and Computational Efficiency.

k	mIoU (%)	Defect mIoU (%)	Inference Time (ms)	Memory consumption (GB)
10	78.86	72.18	25.96	1.37
15	79.74	73.31	28.68	1.50
20	80.32	74.04	31.41	1.62
25	80.04	73.68	34.14	1.75
30	79.46	72.94	36.87	1.87

**Fig 16:**
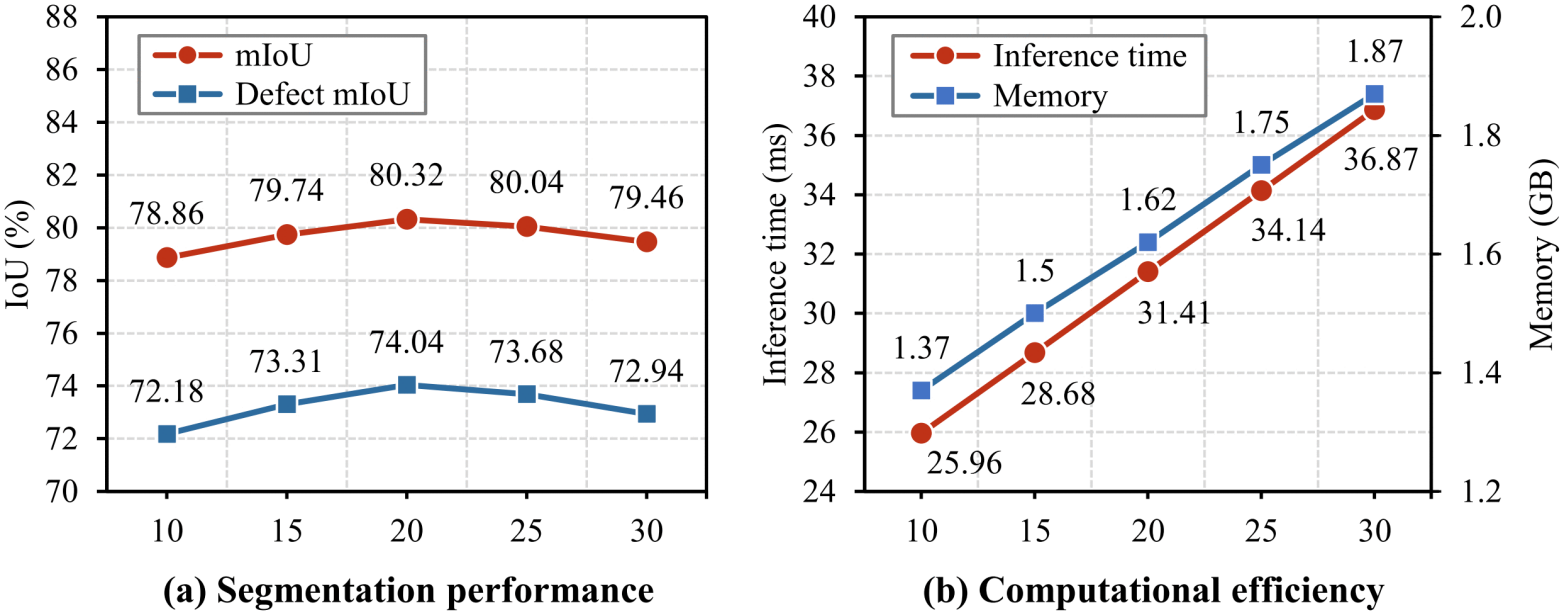
Segmentation Performance and Computational Efficiency under Different Neighborhood Sizes (k).

As shown in Table 8 and Fig 16, increasing k from 10 to 20 progressively improves Overall mIoU and Defect mIoU, which peak at 80.32% and 74.04%, respectively, when k = 20. This indicates that small neighborhoods may provide insufficient coverage of local wire rope geometry, whereas a moderate increase in k enables more complete modeling of defect boundaries and spatial relationships. Performance declines slightly at k = 25 and k = 30, possibly because larger neighborhoods introduce more background points and weaken the geometric distinction between defective and normal regions.

Fig 16a shows that Overall mIoU and Defect mIoU initially increase and then decrease with k, indicating moderate sensitivity to neighborhood size. However, performance remains stable within k = 15–25, where the maximum variation in Overall mIoU is only 0.58 percentage points. Fig 16b shows an approximately linear increase in computational cost: inference time rises from 25.96 to 36.87 ms/sample, while GPU memory consumption increases from 1.37 to 1.87 GB.

At k = 20, the model achieves the highest Overall mIoU and Defect mIoU while maintaining acceptable inference time and memory consumption. Therefore, k = 20 was selected as the default neighborhood size based on segmentation performance, computational efficiency, and parameter robustness. Transformer depth, number of attention heads, and feature dimensionality were fixed across all experiments to ensure controlled comparisons; a more comprehensive analysis of their combinations will be considered in future work.

#### 4.4.6. Multi-seed stability analysis.

To assess the reproducibility of the performance gains and reduce the influence of stochastic training, the baseline model, Point S-GS Former, and WireGC-Former were independently trained and evaluated using random seeds 42, 43, and 44. Point S-GS Former was included because it achieved the best overall segmentation performance among the competing methods. All experimental settings—including dataset splits, input point count, optimizer, learning-rate schedule, training epochs, and evaluation procedure—were kept identical, with only the random seed varied. The results are reported as the mean and sample standard deviation of three independent runs in Table 9.

**Table 9:** Performance Comparison under Different Random Seeds.

Method	Seed	mIoU (%)	Defect mIoU (%)	mAcc (%)
Baseline	42	75.91	68.24	82.54
Baseline	43	75.62	67.88	82.21
Baseline	44	75.79	68.10	82.43
Baseline	Mean ± Std	75.77 ± 0.15	68.07 ± 0.18	82.39 ± 0.17
Point S-GS Former	42	80.18	73.89	85.21
Point S-GS Former	43	79.91	73.55	84.98
Point S-GS Former	44	80.07	73.76	85.12
Point S-GS Former	Mean ± Std	80.05 ± 0.14	73.73 ± 0.17	85.10 ± 0.12
WireGC-Former	42	80.32	74.04	85.63
WireGC-Former	43	80.51	74.27	85.78
WireGC-Former	44	80.39	74.15	85.69
WireGC-Former	Mean ± Std	80.41 ± 0.10	74.15 ± 0.12	85.70 ± 0.08

As shown in Table 9, WireGC-Former consistently outperforms the baseline model across all random seeds. Based on the mean results, it improves mIoU, Defect mIoU, and mAcc by 4.64, 6.08, and 3.31 percentage points, respectively, confirming that the proposed local geometric modeling, feature fusion, and global dependency modeling strategies provide reproducible performance gains rather than improvements specific to a single run. WireGC-Former also maintains a modest advantage over Point S-GS Former for all three metrics under seeds 42, 43, and 44. On average, the improvements in mIoU, Defect mIoU, and mAcc are 0.36, 0.42, and 0.60 percentage points, respectively, demonstrating consistent gains over the strongest competing method.

Overall, WireGC-Former demonstrates stable and reproducible improvements over the baseline model and consistently outperforms Point S-GS Former across different random initializations, further supporting its effectiveness and reliability.

### 4.5. Discussion on the impact of industrial applications

The industrial applicability of the proposed method was assessed in terms of performance, efficiency, and reliability. On WireRope3D, the GCN–Transformer network achieves an Overall mIoU of 80.32%, a Defect mIoU of 74.04%, and an mAcc of 85.63%, outperforming PointNet++, DGCNN, and Point Transformer overall. These results demonstrate its capability for accurate point-level defect segmentation, supporting subsequent defect-boundary localization and geometric parameter analysis. However, its IoU for wire protrusion is 74.04%, lower than the 77.96% achieved by Point Transformer, indicating limitations in segmenting slender defects with ambiguous boundaries and geometries similar to normal strand textures. Because WireRope3D differs from general-purpose public datasets in point cloud structure, defect scale, and class definition, these results primarily reflect task-specific performance on wire rope surface defect segmentation.

In terms of speed, the proposed method has 2.02 M parameters and 3.29 G FLOPs. Although the parameter size is within an acceptable range, the computational cost is still higher than that of some comparison models, indicating that the method is more suitable for periodic offline inspection or high‑precision analysis scenarios, while its deployment in real‑time online inspection still faces certain challenges. In terms of reliability, the method achieves relatively stable segmentation results for the three defect types—wear, wire protrusion, and broken wire—with particularly better performance on broken wire. However, the current experimental data are mainly collected in a laboratory environment; factors such as vibration, occlusion, oil contamination, and strong reflections in complex field conditions may further affect point cloud quality and model performance. Therefore, overall, the proposed method has good potential for industrial application, but further optimization is still needed in weak‑anomaly defect recognition, real‑time performance, and generalization ability under complex operating conditions.

## 5. Conclusion

To address the issues of the lack of industrial product samples in public datasets for point cloud segmentation and the poor performance of small-target defect detection, this paper independently constructs a data acquisition platform based on a 3D laser profile sensor. The system collects point cloud data covering three typical surface defects of wire ropes—wear, wire protrusion, and broken wire—providing important data support for research on deep learning-based defect detection methods. Furthermore, this paper proposes a 3D point cloud segmentation method that integrates a graph convolutional network and a Transformer mechanism to improve the accurate recognition of small-target defects and the capture of global dependencies. Experimental results show that the proposed method achieves significant improvements over traditional networks on the WireRope3D dataset, particularly excelling in segmentation accuracy for broken wire categories. Our WireRope3D dataset fills the gap of lacking industrial product samples in point cloud segmentation tasks, and the proposed method effectively enhances the accuracy of segmentation tasks. Future work includes exploring model lightweight design strategies and expanding data acquisition conditions to increase dataset diversity and model generalization capability.
